# Comparative Analysis of the Diagonal Stride Technique during Roller Skiing and On-Snow Skiing in Youth Cross-Country Skiers †

**DOI:** 10.3390/s24051412

**Published:** 2024-02-22

**Authors:** Mujia Ma, Shuang Zhao, Ting Long, Qingquan Song, Hans-Christer Holmberg, Hui Liu

**Affiliations:** 1 School of Sport Science, Beijing Sport University, Beijing 100084, China; mamujia5683@gmail.com; 2 China Institute of Sport and Health Science, Beijing Sport University, Beijing 100084, China; 3 Dalian Fast Move Technology Co., Ltd., Dalian 116033, China; 4 Auckland Bioengineering Institute, University of Auckland, Auckland 1010, New Zealand; 5 School of Strength and Conditioning Training, Beijing Sport University, Beijing 100084, China; 6 Department of Health Sciences, Mid Sweden University, 831 25 Östersund, Sweden

**Keywords:** technical analysis, preseason training, uphill technique, transfer training

## Abstract

Roller skiing is one primary form of training method as it is an off-snow equivalent to cross-country (XC) skiing during the dry land preseason training, but the results could only be applied to on-snow skiing with appropriate caution. The aim of this present study was to investigate the similarities and differences in roller skiing and on-snow skiing with the diagonal stride (DS) technique. Six youth (age: 14.3 ± 2.9 years) skiers participated in this study. Two high-definition video camcorders and FastMove 3D Motion 2.23.3.3101 were used to obtain the three-dimensional kinematic data. The cycle characteristics and joint angle ROM of the DS technique while skiing on different surfaces were similar. Almost all joint angle–time curves that were obtained from roller skiing showed a moderate-to-high degree of similarity to the angle–time curves obtained from on-snow skiing, except the hip adduction–abduction angle. The differences between roller skiing and on-snow skiing were mainly found in the body and calf anteversion angles, and the joint angles at critical instants. DS roller skiing can simulate DS on-snow skiing to a large extent in youth athletes. The hip movement, knee flexion, and calf anteversion at ski/roller ski touchdown and take-off, pole inclination at pole touchdown, body anteversion angle, and trunk anteversion angle at pole touchdown were the points that required caution when transferring preseason practice roller skiing to on-snow skiing.

## 1. Introduction

Cross-country (XC) skiing is an endurance sport which is performed in varying terrain on snow [1,2]. For most skiers, roller skiing is one primary form of training method as it is an off-snow equivalent to XC skiing during the dry land preseason training months (May to October) of the year [3,4]. Roller skiing is also a ski-specific laboratory testing model that is often used in the research field [5]. Most of the biomechanical studies [6,7,8,9,10] were performed in a controlled environment with roller skiing in the laboratory; however, the results could only be applied to on-snow skiing with appropriate caution [11].

Although strong correlations were found between treadmill roller skiing [12], laboratory-based test [3], and on-snow skiing performance, the differences between roller skiing and on-snow skiing have been investigated from different aspects. For example, skiers train nearly half of the year on different types of roller skis which are normally heavier than the skis used in on-snow skiing. The increased ski weight has been proven to affect the metabolic cost and gross efficiency during steep uphill roller skiing in the V1 skating technique [13]. While skiing on snow, several factors may influence the static and dynamic friction coefficients of the skis, which may further affect the skiing technique. The dynamic friction coefficient when XC skis glide on snow has been reported in different studies to range from 0.02 to 0.18 [14,15,16]. While roller skiing, previous studies have shown that the dynamic friction coefficient for roller skis ranged from 0.011 to 0.026 [17,18]. In addition, studies have shown that the static friction coefficient (µ_s_) of roller skis on a treadmill rubber mat and dry and wet asphalt surfaces is 5–8 times higher than that of skis on snow [19,20], which may cause differences in movement patterns when skiing on different surfaces [21]. The kinematic differences between uphill roller skiing and on-snow roller skiing using the V2 skating technique have been revealed recently. V2 roller ski skating can well simulate on-snow ski skating, but the changing skiing surface may alter the skier’s hip movements and pole push times [22]. However, the kinematics comparison between roller skiing and on-snow skiing has not been performed by using classic style skiing techniques in XC skiing.

The diagonal stride (DS) is one of the sub-techniques in classical XC skiing [1,23,24]. During this technique, the arms and legs follow a diagonal pattern to generate the propulsive force [25]. The alternation of ski and pole propulsion by the left and right body sides allows an almost-continuous propulsion action during the DS [25,26]; thus, the DS is the major and preferred sub-technique at steep inclines [1]. In long-distance classical XC skiing, the uphill speed is thought to have the highest relevance to skiers’ success [27,28,29]. The Rating of Perceived Exertion (RPE) and percentages of technique-specific VO_2peak_ of the DS were lower than those of other classic style sub-techniques [30]. These also emphasized the importance of the DS technique.

Consequently, the aim of the present study was to investigate the similarities and differences in roller skiing and on-snow skiing with the DS technique. As the performance of the DS has been proven to correlate with cycle characteristics (e.g., cycle length, leg swing time, and gliding time) and joint kinematics (e.g., hip flexion range of motion) [26], both cycle characteristics and joint kinematics were involved in this comparative analysis. According to the effect of skiing surface on the V2 skating technique [22], we hypothesized that similarities and differences between roller skiing and on-snow skiing would be found in cycle characteristics as well as joint kinematics with the DS technique with youth skiers.

## 2. Methods

### 2.1. Subjects

Six male youth XC skiers (age = 14.3 ± 2.9 years; height = 165.7 ± 13.1 cm; weight = 48.6 ± 11.0 kg) participated in this study. All subjects were members of the youth XC ski team of Jilin Province, China. All subjects had at least 2 years of experience in performing the DS technique and were familiar with roller skiing. Ethical approval for this study was obtained from the Sports Science Experiment Ethics Committee of Beijing Sport University (approval number 2020187H). All subjects gave written informed consent, and parental consent was provided for athletes younger than 18 years old (*n* = 5).

### 2.2. Overall Design

The measurements were performed twice under different environmental conditions in 2021. The asphalt roller skiing test was completed in May at the National Roller Skiing Base in Jingyuetan National Forest Park, Changchun City, Jilin Province, China. The on-snow skiing test was completed in July, in the ski tunnel at the Beishan Four Seasons XC Ski Resort in Jilin City, Jilin Province, China. As slopes with the same degree were not found at these two places, the 7.6° and 6.6° uphill slopes were chosen for the asphalt roller skiing and on-snow skiing tests, respectively.

The subjects wore traditional ski boots (Alpina, Žiri, Slovenia), poles (One Way, Vantaa, Finland), and roller skis (Karhu, Helsinki, Finland) with a front ratchet for the roller skiing test and skis (Fischer, Ried im Innkreis, Austria) for the on-snow skiing test. The subjects started using the DS at the starting point and passed through the test slope at maximum speed, before returning to the starting point for the second trial. Each subject completed two trials of the test by performing the DS at maximum speed within the test slope. The starting point was on the flat ground about 10 m away from the test slope. The average of the two trials in each test was used for further analysis.

### 2.3. Data Collection

Two high-definition video camcorders (SONY FDR-AX700, SONY Corporation, Tokyo, Japan) were used to record the roller skiing and on-snow skiing DS technique of each subject at 60 fps with a shutter speed of 1/500 s. Two cameras were placed on the left and right sides of the test slopes. The angle between the optical axes of the two cameras was approximately 85° to ensure that as many body landmarks of interest as possible remained in view of the two cameras for the duration of the DS cycle.

**Figure 1:**
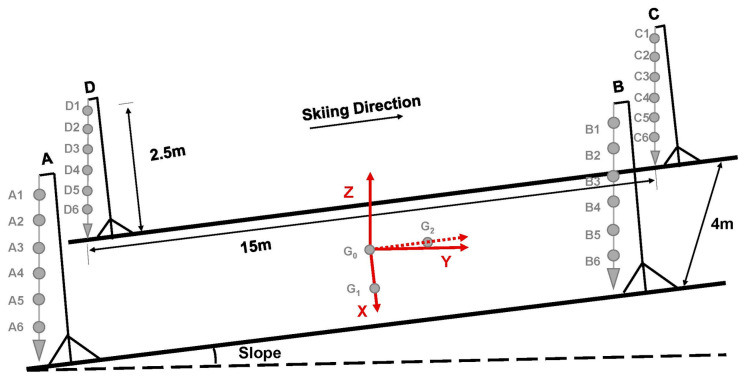
Calibration set up for data collection and global coordinate system setting. (Note: A, B, C, and D represent the four pillars. A1 to A6, B1 to B6, C1 to C6, and D1 to D6 represent the small balls that hung on strings. G_1_, G_2_, and G_3_ represent the markers which were placed in the center of the calibration space. The red arrows represent the X, Y, and Z axis of the global coordinate system. The skiing direction is along the roller skiing or on-snow skiing slope).

Four pillars which hung a string of six small balls were set up for calibration. The distance between the centers of the ground projections of the four sets of balls was measured. The four sets of balls formed a 15 m long × 4 m wide × 2.5 m high calibration space. The calibration space was located about 10 m after entering the test slope and could cover at least three DS cycles. Three markers were placed in the center of the calibration space to establish a global coordinate system. The X-axis pointed toward the right side, the Y-axis pointed to the horizontal direction, and the Z-axis pointed upward along with the line of gravity (Figure 1). The X-axis, Y-axis, and Z-axis were orthometric. The skiing direction was along the roller skiing or on-snow skiing slopes. A similar set up for calibration and the global coordinate system can be found elsewhere [31,32]. For both the roller skiing test and on-snow test, the two cameras were synchronized by using a flash.

### 2.4. Data Reduction

**Figure 2:**
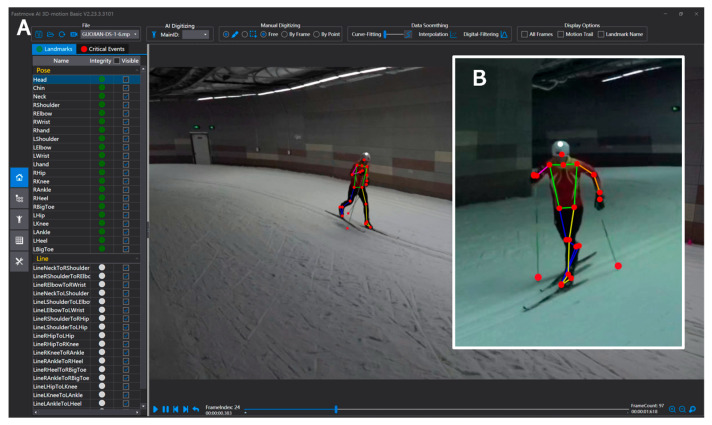
(**A**) One video clip from the camcorder located on the right side of the skiing direction and the interface of FastMove 3D Motion software. (**B**) One video clip from the camcorder located on the left side of the skiing direction. (Note: Figures shown in both (**A**) and (**B**) were the same moment from one trail of one subject. The red points were the landmarks recognized automatically by FastMove 3D Motion).

The mean resultant calibration error (the distance between the estimated position and the known position) was lower than 10 mm. The video clips of each trial of each subject from both camcorders were automatically digitized using the FastMove 3D Motion (FastMove 3D Motion 2.23.3.3101, FastMove Technology, Inc., Dalian, China) to obtain two-dimensional (2D) coordinate data. For each trial, 21 critical body landmarks [31,32,33] and the left and right pole tips were digitized in each frame (Figure 2). The critical body landmarks included the top of the head, the center point of the transverse section of the head, the midpoint of the left and right shoulder joint center, bilateral joint centers of six joints (wrist, elbow, shoulder, hip, knee, and ankle), the bilateral distal point of the third metacarpal bone, bilateral heel points, and bilateral big toe points. Real-life three-dimensional (3D) coordinates of the 21 body landmarks and two pole points (one on each pole) were estimated from synchronized 2D coordinates using the direct linear transformation method [34]. Estimated real-life 3D coordinates were filtered through a Butterworth low-pass digital filter at a cutoff frequency of 10 Hz.

In order to reduce the impact of the lens distortion on the recorded images, the complete DS cycles near the middle of the image were chosen for analysis. A complete cycle of a DS involves both the leg cycle and pole cycle. One cycle from each trial was analyzed. The leg cycle starts with the right ski/roller ski touchdown and ends with the subsequent right ski/roller ski touchdown. It consists of two phases: the stance phase, which begins with the right ski/roller ski touchdown and ends with the take-off, and the swing phase, which starts with the ski/roller ski take-off and ends with the next right ski/roller ski touchdown. The leg cycle time (LCT) is the period of the leg cycle. The leg frequency (LF) is the leg cycles per second (LF = 1/LCT). The stance phase time (ST) and the swing phase time (SWT) are the durations of the corresponding phases. The stance phase relative time (ST%) and the swing phase relative time (SWT%) are the ST and SWT as the percentage of the LCT, respectively. The leg cycle length (LCL) is the distance between the consecutive right ski/roller ski touchdown. The leg speed (LS) is represented by the average speed during one leg cycle (LS = LCL/LCT).

The pole cycle begins with the right pole touchdown and ends with the subsequent right pole touchdown. It consists of two phases: the poling phase, which starts with the right pole touchdown and ends with the right pole take-off, and the recovery phase, which begins with the right pole take-off and ends with the next right pole touchdown. The pole cycle time (PCT) is the period of the pole cycle. The pole frequency (PF) is the pole cycles per second (PF = 1/PCL). The poling time (PT) and the recovery time (RT) are the periods of the corresponding phases. The poling phase relative time (PT%) and the recovery phase relative time (RT%) are the PT and RT as the percentage of the PCT, respectively. The pole cycle length (PCL) is the distance between consecutive right pole touchdowns. The pole speed (PS) is represented by the average speed during one pole cycle (PS = PCL/PCT).

**Figure 3:**
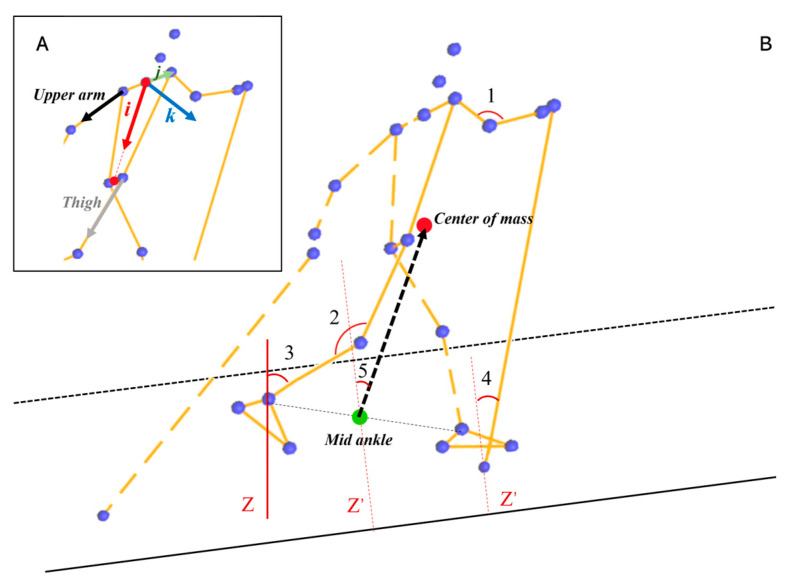
Illustration of the trunk reference frame and the examined joint angles. (**A**) Illustration of the trunk reference frame. (Note: The red, green, and blue arrows are the *i*, *j*, and *k* coordinate axes of the trunk reference frame, respectively. The black and gray arrows are the upper arm and thigh vectors, respectively). (**B**) Illustration of the examined joint angles. 1, 2, 3, 4, and 5 represent the elbow flexion–extension angle, knee flexion–extension angle, calf anteversion angle, pole inclination, and the body anteversion angle, respectively. (Note: The red point is the center of mass. The green point represents the midpoint of the right and left ankle joint center. The black dashed arrow is the vector from the midpoint of the right and left ankle joint center to the center of mass. The dotted line Z’ is the vertical vector pointing upwards and perpendicular to the slope. The red line is the Z axis of the global coordinate system which is along the line of gravity).

The shoulder, elbow, hip, knee, calf anteversion, body anteversion, trunk anteversion, and pole inclination angles were deduced for analysis. The shoulder/hip angles were calculated by projecting the angles between the upper arm/thigh vector and the longitudinal axis of the trunk reference frame to the corresponding planes in the trunk reference frame. The upper arm vector was defined by using the right shoulder joint center and the right elbow joint center (Figure 3A). The thigh vector was defined by using the right hip joint center and the right knee joint center (Figure 3A). The trunk reference frame (*i, j, k*) was defined by using the midpoint of the left and right shoulder joint center, the left shoulder joint center, and the midpoint of the left and right hip joint center (Figure 3A). The flexion–extension and adduction–abduction angles were the projected angles on the sagittal plane (*ik* plane) and coronal plane (*ij* plane) of the trunk reference frame, respectively. The elbow flexion–extension angle was defined as the angle between the vector pointing from the right elbow joint center to the right shoulder joint center and the vector pointing from the right elbow joint center to the right wrist center (Figure 3B). The knee flexion–extension angle was defined as the angle between the vector pointing from the right knee joint center to the right hip joint center and the vector pointing from the right knee joint center to the right ankle joint center (Figure 3B). The body anteversion angle is defined as the angle between the vector pointing from the midpoint of the two ankles to the body center of mass and the vertical vector that is perpendicular to the slope (Figure 3B). The calf anteversion angle is defined as the angle between the vector pointing from the right ankle to the right knee point and the Z-axis (Figure 3B). The pole inclination is defined as the angle between the vector pointing from the right pole point to the right hand and the vertical vector that is perpendicular to the slope (Figure 3B).

The hip flexion–extension angle, hip adduction–abduction angle, knee flexion–extension angle, calf anteversion angle, body anteversion angle, and trunk anteversion angle at right ski/roller ski touchdown and take-off were analyzed during the leg cycle. The shoulder flexion–extension angle, shoulder adduction–abduction angle, elbow flexion–extension angle, body anteversion angle, trunk anteversion angle, and pole inclination at right pole touchdown and take-off were analyzed during the pole cycle. For both leg and pole cycle, the range of motion (ROM) of each analyzed angle was calculated as well.

### 2.5. Statistical Analyses

The coefficient of multiple correlation (CMC) was calculated to assess the similarity in angle–time curves of DS techniques between roller skiing and on-snow skiing in each phase [35,36,37,38]. The CMC results range from 0 to 1; values between 0 and 0.25 indicate no similarity between curves; values between 0.25 and 0.50 indicate a low degree of similarity; values between 0.50 and 0.75 indicate a moderate degree of similarity; and values greater than or equal to 0.75 suggest a high degree of similarity [39]. The CMC calculation was carried out in Microsoft Excel 2019 (Microsoft Corporation, Redmond, WA, USA).

The Wilcoxon signed rank test was performed to compare the cycle characteristics and the joint kinematics of DS techniques between roller skiing and on-snow skiing. The one-dimensional statistical parametric mapping (SPM) paired sample *t*-test program was performed to compare the differences in angle–time curves of DS techniques between roller skiing and on-snow skiing in each phase. The SPM analyses were accomplished using the open-source spm1d code (M.0.4.9, www.spm1d.org). Both the Wilcoxon signed rank test and SPM analyses were performed in MATLAB R2020a (MathWorks, Natick, MA, USA). The level of statistical significance was set at 0.05. 

## 3. Results

### 3.1. Cycle Characteristics

**Figure 4:**
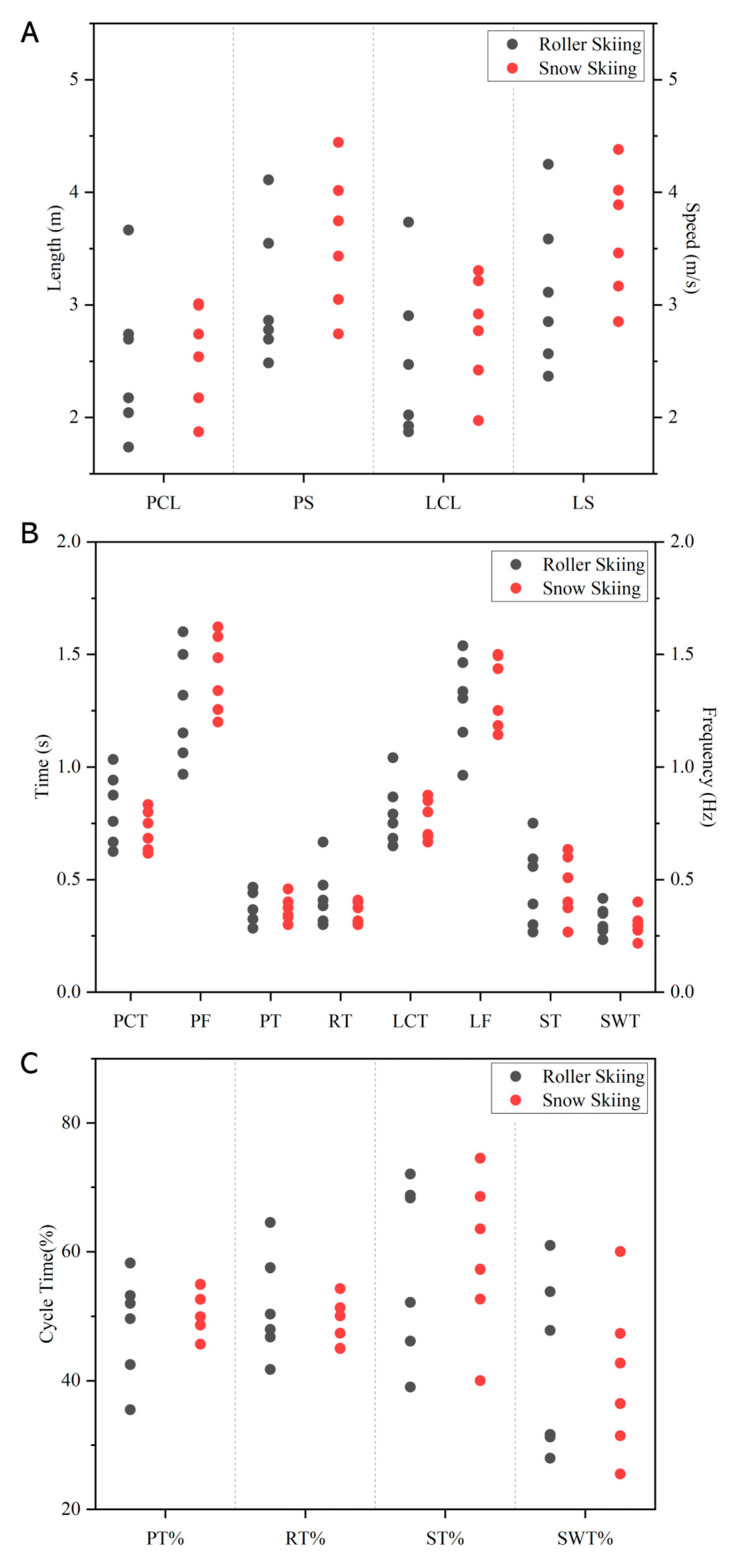
Comparison of cycle characteristics between roller skiing and on-snow skiing. (**A**) Cycle length (left axis) and cycle speed (right axis). PCL, PS, LCL, and LS represent the pole cycle length, pole speed, leg cycle length, and leg speed, respectively. (**B**) Cycle time (left axis), phase time (left axis), and cycle frequency (right axis). The PCT, PF, PT, and RT represent the pole cycle time, poling frequency, poling time, and recovery time, respectively. The LCT, LF, ST, and SWT represent the leg cycle time, leg frequency, leg stance time, and leg swing time, respectively. (**C**) Phases relative time. PT%, RT%, ST%, and SWT% represent the relative poling time, relative recovery time, relative leg stance time, and relative leg swing time. The data are presented as subject’s personal data. Each round point represents one subject. The black round points represent data from roller skiing. The red round points represent data from on-snow skiing.

No significant difference was found in the cycle characteristics of DS techniques between roller skiing and on-snow skiing (*p* ≥ 0.310, Figure 4). During the pole cycle, the maximum speed was 3.08 ± 0.57 m/s and 3.57 ± 0.57 m/s, respectively. During the leg cycle, the maximum speed was 3.12 ± 0.64 m/s and 3.62 ± 0.52 m/s, respectively. No significant difference was found in maximum speed between roller skiing and on-snow skiing in both leg and pole cycle (*p* = 0.240, *p* = 0.180).

### 3.2. Joint Kinematics of Leg Cycle

**Figure 5:**
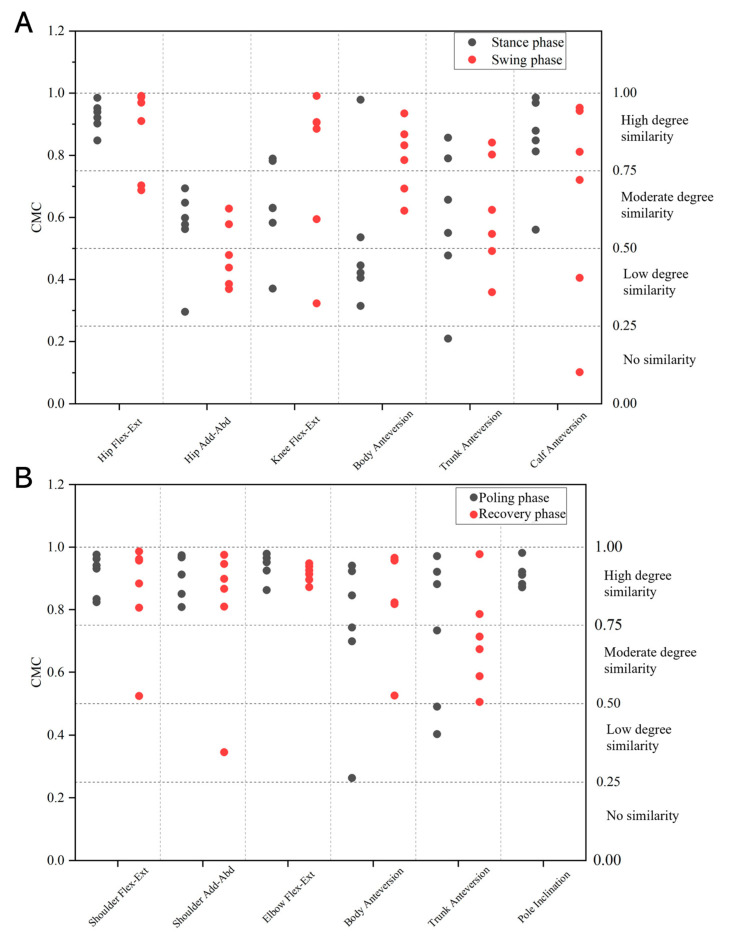
The similarities (CMC values) of angle–time curves between roller skiing and on-snow skiing during leg cycle and pole cycle. (**A**) The CMC values during leg cycle. The black round points represent the CMC values from leg stance phase. The red round points represent the CMC values from leg swing phase. (**B**) The CMC values during pole cycle. The black round points represent the CMC values from poling phase. The red round points represent the CMC values from recovery phase. The data are presented as subject’s personal data. Each point represents one subject.

During the leg stance phase, the angle–time curves of hip flexion–extension and calf anteversion angles in the roller skiing test exhibited a high degree of similarity when compared to the angle–time curves in the on-snow skiing test (CMC: 0.925 ± 0.043, 0.843 ± 0.140; Figure 5A). The angle–time curves of the hip adduction–abduction angle, knee flexion–extension angle, body anteversion angle, and trunk anteversion angle in the roller skiing test showed a moderate degree of similarity when compared to the angle–time curves in the on-snow skiing test (CMC: 0.563 ± 0.139, 0.631 ± 0.154, 0.536 ± 0.260, 0.551 ± 0.238; Figure 5A). During the swing phase, the angle–time curves of the hip and knee flexion–extension angles and the body anteversion angle while roller skiing showed a high degree of similarity when compared to on-snow skiing (CMC: 0.875 ± 0.130, 0.768 ± 0.235, 0.832 ± 0.102; Figure 5A). However, a low degree of similarity was observed in the hip adduction–abduction angle (CMC: 0.479 ± 0.109; Figure 5A). The angle–time curves of trunk and calf anteversion angles while roller skiing showed a moderate degree of similarity (CMC: 0.635 ± 0.176, 0.656 ± 0.308; Figure 5A).

**Figure 6:**
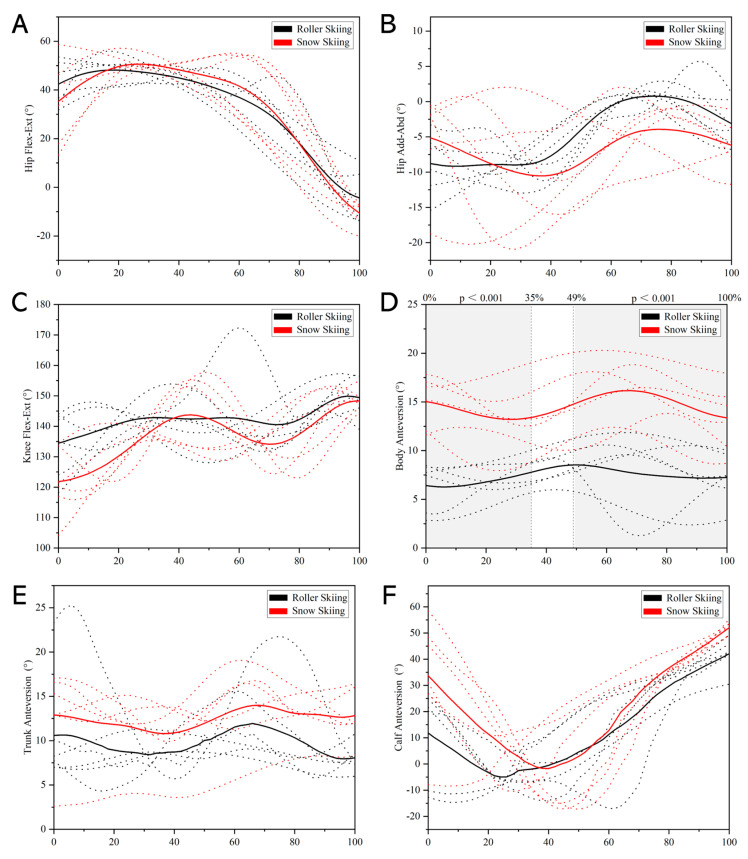
Comparison of leg cycle joint kinematics during leg stance phase between roller skiing and on-snow skiing. (**A**) Angle–time curves of hip flexion–extension angle. (**B**) Angle–time curves of hip adduction–abduction angle. (**C**) Angle–time curves of knee flexion–extension angle. (**D**) Angle–time curves of body anteversion angle. (**E**) Angle–time curves of trunk anteversion angle. (**F**) Angle–time curves of calf anteversion angle. (Note: The dotted lines represent the angle–time curves during stance phase from each subject. The solid lines represent the average data of all subjects. The colors black and red represent roller skiing and on-snow skiing, respectively. Areas under the gray shade were the parts where a significant difference between roller skiing and on-snow skiing was found).

During the stance phase, the body anteversion angle during the on-snow skiing test was significantly greater than that during the roller skiing test in 0–35% and 49–100% of the stance phase (*p* < 0.001, Figure 6D). However, no significant difference was found in the hip flexion–extension angle, hip adduction–abduction angle, knee flexion–extension angle, trunk anteversion angle, and calf anteversion angle between roller skiing and on-snow skiing (Figure 6).

**Figure 7:**
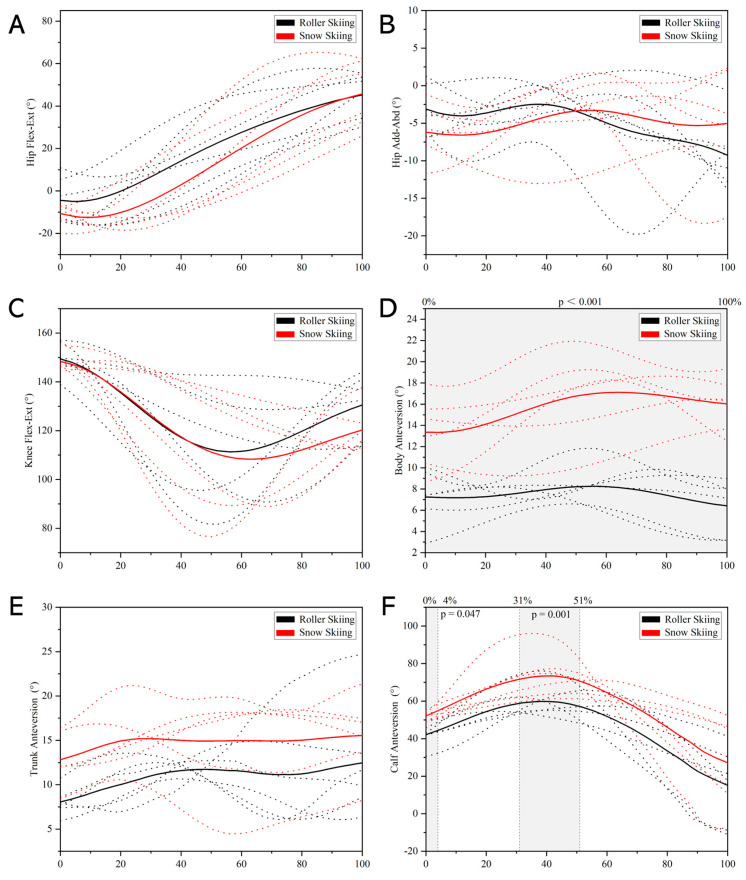
Comparison of leg cycle joint kinematics during leg swing phase between roller skiing and on-snow skiing. (**A**) Angle–time curves of hip flexion–extension angle. (**B**) Angle–time curves of hip adduction–abduction angle. (**C**) Angle–time curves of knee flexion–extension angle. (**D**) Angle–time curves of body anteversion angle. (**E**) Angle–time curves of trunk anteversion angle. (**F**) Angle–time curves of calf anteversion angle. (Note: The dotted lines represent the angle–time curves during stance phase from each subject. The solid lines represent the average data of all subjects. The colors black and red represent roller skiing and on-snow skiing, respectively. Areas under the gray shade were the parts where a significant difference between roller skiing and on-snow skiing was found).

During the swing phase, the body anteversion angle while on-snow skiing was significantly greater than that when roller skiing (*p* < 0.001, Figure 7D). The calf anteversion angle during 0–4% (*p* = 0.047) and 31–51% (*p* = 0.001) of the swing phase while on-snow skiing was significantly greater than that when roller skiing (Figure 7F). However, no significant difference was found in the hip flexion–extension angle, hip adduction–abduction angle, knee flexion–extension angle, and trunk anteversion angle between roller skiing and on-snow skiing (Figure 7).

During the leg stance phase and leg swing phase, a significant difference was not found in any joint’s ROM between roller skiing and on-snow skiing (*p* ≥ 0.093, Table 1).

The knee flexion angle at roller ski touchdown while roller skiing was significantly greater than the knee flexion angle at ski touchdown while on-snow skiing (*p* = 0.041, Table 2). The body and calf anteversion angles were significantly greater at ski/roller ski touchdown while on-snow skiing than roller skiing (*p* = 0.002, *p* = 0.041, Table 2). There was no significant difference in hip flexion angle, hip adduction–abduction angle, and trunk anteversion angle between roller skiing and on-snow skiing (*p* ≥ 0.132, Table 2). At ski/roller ski take-off, the body, trunk, and calf anteversion angles while on-snow skiing were significantly greater than those angles while roller skiing (*p* ≤ 0.009, Table 2). And no difference was found in hip flexion–extension, hip adduction–abduction, and knee flexion angle between roller skiing and on-snow skiing at ski/roller ski take-off (*p* ≥ 0.180, Table 2). 

### 3.3. Joint Kinematics of Pole Cycle

During the poling phase, the angle–time curves of the shoulder flexion–extension angle, shoulder adduction–abduction angle, elbow flexion–extension angle, and pole inclination while roller skiing showed a high degree of similarity when compared to the corresponding angle–time curves while skiing on snow (CMC: 0.911 ± 0.060, 0.914 ± 0.064, 0.941 ± 0.039, and 0.912 ± 0.050; Figure 5B). The angle–time curves of body and trunk anteversion angles in the roller skiing test showed a moderate degree of similarity when compared to the angle–time curves in the on-snow skiing test (CMC: 0.743 ± 0.279, 0.734 ± 0.237; Figure 5B). During the recovery phase, the angle–time curves of the trunk anteversion angle while roller skiing showed a moderate degree of similarity when compared to on-snow skiing (CMC: 0.714 ± 0.183; Figure 5B). The angle–time curves of the shoulder flexion–extension angle, shoulder adduction–abduction angle, elbow flexion–extension angle, and body anteversion angle while roller skiing showed a high degree of similarity when compared to the corresponding angle–time curves while skiing on snow (CMC: 0.853 ± 0.1591, 0.807 ± 0.213, 0.916 ± 0.026, and 0.818 ± 0.178; Figure 5B). 

**Figure 8:**
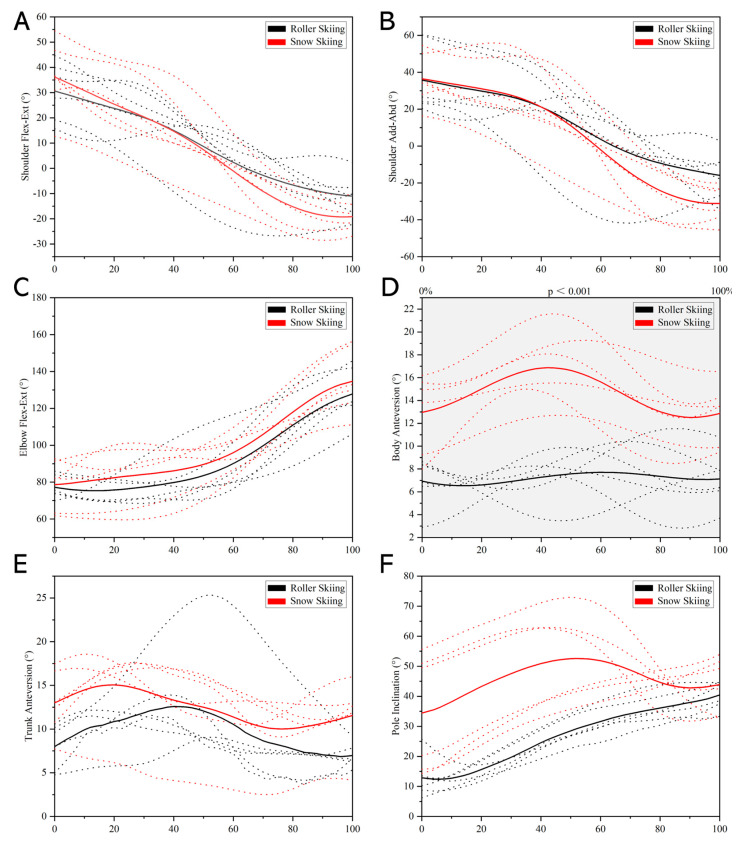
Comparison of pole cycle joint kinematics during poling phase between roller skiing and on-snow skiing. (**A**) Angle–time curves of shoulder flexion–extension angle. (**B**) Angle–time curves of shoulder adduction–abduction angle. (**C**) Angle–time curves of elbow flexion–extension angle. (**D**) Angle–time curves of body anteversion angle. (**E**) Angle–time curves of trunk anteversion angle. (**F**) Angle–time curves of pole inclination. (Note: The dotted lines represent the angle–time curves during stance phase from each subject. The solid lines represent the average data of all subjects. The colors black and red represent roller skiing and on-snow skiing, respectively. Areas under the gray shade were the parts where a significant difference between roller skiing and on-snow skiing was found).

**Figure 9:**
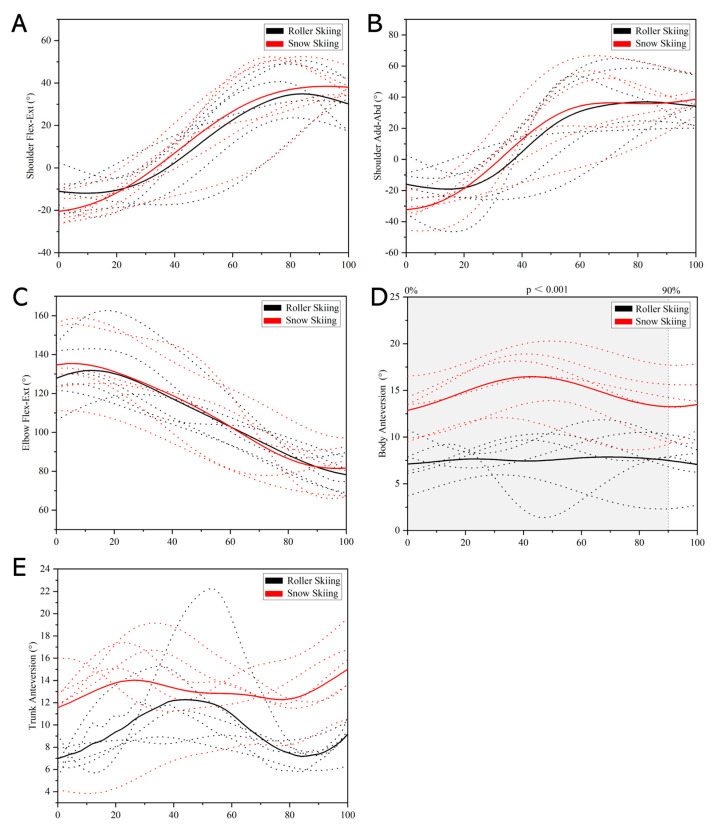
Comparison of pole cycle joint kinematics during recovery phase between roller skiing and on-snow skiing. (**A**) Angle–time curves of shoulder flexion–extension angle. (**B**) Angle–time curves of shoulder adduction–abduction angle. (**C**) Angle–time curves of elbow flexion–extension angle. (**D**) Angle–time curves of body anteversion angle. (**E**) Angle–time curves of trunk anteversion angle. (Note: The dotted lines represent the angle–time curves during stance phase from each subject. The solid lines represent the average data of all subjects. The colors black and red represent roller skiing and on-snow skiing, respectively. Areas under the gray shade were the parts where a significant difference between roller skiing and on-snow skiing was found).

During the poling phase, the body anteversion angle during the on-snow skiing test was significantly greater than that during the roller skiing test in the whole stance phase (*p* < 0.001, Figure 8D). However, no significant difference was found in the shoulder flexion–extension angle, shoulder adduction–abduction angle, elbow flexion–extension angle, trunk anteversion angle, and pole inclination between roller skiing and on-snow skiing (Figure 8). During the recovery phase, the body anteversion angle during 0–90% of the recovery phase while on-snow skiing was significantly greater than that when roller skiing (*p* < 0.001, Figure 9D). However, no significant difference was found in the shoulder flexion–extension angle, shoulder adduction–abduction angle, elbow flexion–extension angle, and trunk anteversion angle between roller skiing and on-snow skiing (Figure 9).

During both poling and recovery phases, the joint ROM of the shoulder flexion–extension angle, shoulder adduction–abduction angle, elbow flexion–extension angle, body anteversion angle, trunk anteversion angle, and pole inclination established no significant difference between roller skiing and on-snow skiing (*p* ≥ 0.093, Table 3).

At pole touchdown, the body and trunk anteversion angles and the pole inclination when skiing on snow were significantly greater than that when roller skiing (*p* ≤ 0.026, Table 4). No significant difference was found in the shoulder flexion angle, shoulder abduction angle, and elbow flexion angle at pole touchdown between roller skiing and on-snow skiing (*p* ≥ 0.818, Table 4). At pole take-off, the shoulder adduction–abduction angle and the body anteversion angle in the on-snow skiing test was greater than that in the roller skiing test (*p* ≤ 0.041, Table 4). No significant difference was found in the shoulder flexion–extension angle, elbow flexion angle, trunk anteversion angles, and the pole inclination at pole touchdown between roller skiing and on-snow skiing (*p* ≥ 0.818, Table 4). 

## 4. Discussion

The current study aimed to investigate the similarities and differences in roller skiing and on-snow skiing by using the DS technique with youth skiers. It was expected that similarities and differences between roller skiing and on-snow skiing would be found in cycle characteristics as well as joint kinematics with the DS technique with youth skiers. These expectations were mostly confirmed by the results of this study. Our main findings were as follows: (1) the cycle characteristics of the DS technique were not affected by the skiing surface; (2) the joint angles’ ROM of the DS technique while skiing on different surfaces was similar; (3) almost all joint angle–time curves that were obtained from roller skiing showed a moderate-to-high degree of similarity to the angle–time curves obtained from on-snow skiing, except the hip adduction–abduction angle; (4) the differences between roller skiing and on-snow skiing were mainly found in the body and calf anteversion angles, and the joint angles at critical instants.

In this study, the cycle characteristics and the angles’ ROM of the DS technique while roller skiing had no difference when compared with skiing on snow. Previous studies indicated that the cycle length, leg swing time, gliding time, and hip flexion ROM have been proven to correlate with the performance of the DS [26], but the results could only be applied to on-snow skiing with appropriate caution [11]. The uphill slopes for the roller skiing and on-snow skiing tests in the current study were different. Previous studies have shown that the cycle characteristics of the DS technique [40] and other skiing techniques [41,42] were affected by the increasing incline. The results of this study are different from previous results, which may be due to the fact that the skiing speed was not strictly controlled in this study. Moreover, this may also relate to the fact that the two tests were conducted at maximum DS skiing speed. Whether the same results would be achieved when performing the measurement at the same sub-maximum speed (or competition speed) needs further investigation. 

Except for the angle–time curves of the hip adduction–abduction angle, the angle–time curves of almost all joint angles that were obtained from roller skiing showed a moderate-to-high degree of similarity to the angle–joint curves obtained from on-snow skiing. In this study, the similarity between angle–joint curves while skiing on different surfaces was quantified by using the CMC. The CMC values for the lower limb angles (except the hip angle), body anteversion angle, and trunk anteversion angle ranged from 0.536 to 0.925. The CMC values for the upper limb angle and pole inclination ranged from 0.807 to 0.941. The CMC is a value ranging from 0 to 1, which depicts the similarity between waveforms [35,36]. A CMC value close to 1 indicates that the curves involved are similar [9,35,38]. Therefore, DS roller skiing can simulate the DS on-snow skiing to a large extent. The CMC value of angle–time curves of the hip adduction–abduction angle was 0.479 ± 0.109 during the leg swing phase, which indicated a low degree of similarity. The hip movement might be altered while skiing on different surfaces.

Although the trend of the body anteversion angle of the DS technique was similar between roller skiing and on-snow skiing, the body anteversion angle while skiing on-snow was significantly greater than that while roller skiing in this study (Figure 6, Figure 7, Figure 8 and Figure 9). In addition, the calf anteversion angle during 0–4% and 31–51% of the leg swing phase while on-snow skiing was significantly greater than that when roller skiing. This may be due to the equipment used in roller skiing and on-snow skiing being different. The extra weight and height of roller skis may increase the instability. Thus, youth skiers may try to keep their balance by decreasing the body anteversion angle during roller skiing. These skis are more flexible and almost three times longer than the roller skis [22], which may be the reason why a significant difference in calf anteversion angle was found during the leg swing phase when skiing on different surfaces.

Differences between roller skiing and on-snow skiing were also found in joint angles at critical instants (i.e., ski/roller ski touchdown and take-off, pole touchdown and take-off). The roller skiing performance with the DS technique and the amount of impulse generated in the leg force during push-off were also associated with a larger ROM extension in the knee and ankle joints [26]. This demonstrates the potential importance of using a combined pronounced knee joint extension and plantar flexion in DS roller skiing to generate high propulsive leg forces during leg joint extension [26,43]. From a muscular point of view, this dynamic and preparatory flexion pattern in the hip, knee, and ankle joints has been described as a crucial part of a stretch-shortening cycle, with the function of pre-loading the corresponding extensor muscles for an efficient generation of ground reaction forces during the following leg thrust. The knee flexion angle at ski touchdown while on-snow skiing was smaller than the knee flexion angle at roller ski touchdown while roller skiing. A smaller knee flexion angle indicted a greater degree of flexion in knee angle (Figure 3), which allowed the plantar flexor muscle group to generate sufficient force and increase the leg push-off force and on-snow impulse. This may indicate that when the youth skiers perform the DS technique, the knee joint flexion movement might be altered to adapt to the different skiing surfaces. The calf anteversion angles at ski touchdown and take-off while on-snow skiing were greater than those at roller ski touchdown and take-off while roller skiing. This may be related to the difference in the static friction coefficient between roller skiing and on-snow skiing. Previous studies indicated that the static friction coefficient of roller skis on different surfaces was 5–8 times greater than that of skis on snow [19,20]. A higher static friction coefficient may allow the skiers to kick straight back in a tangential direction to obtain the propulsive force. Therefore, a smaller calf anteversion angle at ski/roller ski touchdown and take-off was found in roller skiing. According to the result related to the knee joint angle and calf anteversion angle, one possible awareness of transferring the roller ski DS technique to on-snow skiing is that skiers may not be used to the greater calf anteversion as well as the greater degree of knee flexion, and therefore not use all the potential static friction between skis and snow while on-snow skiing. 

Greater pole inclination, body, and trunk anteversion angles were also found in the on-snow skiing test during the poling phase upon pole touchdown (Table 4). A greater pole inclination angle at the moment of landing on snow is used to increase horizontal propulsion force, and a larger body and trunk anteversion angle is used to lower the position of the center of mass. Therefore, when young athletes use roller skiing for dry land DS training, additional training of the upper limb extensor muscle groups may help make the movements in practice more similar to the real on-snow skiing technique. 

Our study has several limitations. Firstly, the subjects involved in this study were youth athletes, whose techniques are still in development. Therefore, the parameters included in this study may have large individual differences. Whether high-level athletes would have the same results is unclear. In addition, the weather condition, snow condition, ski wax, and detailed information about ski equipment were not controlled. These were all factors that may alter the static and dynamic friction coefficients and skier’s physiological demands and further affect the skiing technique. The results of this study could reveal the differences between roller skiing and on-snow skiing, but it is difficult to reveal which of these is the main factor. Moreover, slopes with the same degree were not found at these two testing places, and the inclines of the slopes were different. The roller skiing and on-snow skiing tests were conducted at maximum DS skiing speed and these speeds were not controlled. Whether the same results would be achieved when performing the measurement at the same sub-maximum speed (or competition speed) needs further investigation.

## 5. Conclusions

DS roller skiing can simulate DS on-snow skiing to a large extent in youth athletes. One possible awareness of transferring the roller ski DS technique to on-snow skiing is that skiers may not be used to the greater calf anteversion as well as the greater degree of knee flexion, and therefore not use all the potential static friction between skis and snow. The hip movement, pole inclination at pole touchdown, body anteversion angle, and trunk anteversion angle at pole touchdown were different between roller skiing and on-snow skiing, which were also the points that required caution when transferring preseason dry land practice roller skiing to on-snow skiing. In addition, during the preseason training, the roller skiing practice combined with the training for upper limb extensor muscle groups may help youth skiers transfer the technique practiced by roller skiing to real snow skiing and master the effective technique.

## Figures and Tables

**Table 1:** Comparison of joint ROM in leg cycle between roller skiing and on-snow skiing during stance and swing phases.

ROM	Stance Phase			Swing Phase		
	Roller Skiing	On-Snow Skiing	*p*-Value	Roller Skiing	On-Snow Skiing	*p*-Value
Hip flexion–extension angle (°)	56.9 ± 10.6	67.7 ± 5.2	0.180	51.7 ± 12.0	64.3 ± 8.6	0.240
Hip adduction–abduction angle (°)	16.9 ± 2.4	15.6 ± 3.4	0.589	11.7 ± 3.0	78.3 ± 16.4	0.699
Knee flexion–extension angle (°)	27.8 ± 6.0	36.2 ± 10.3	0.132	43.5 ± 19.5	57.7 ± 16.4	0.589
Body anteversion angle (°)	4.8 ± 1.6	5.1 ± 1.1	0.310	3.6 ± 1.1	4.6 ± 0.9	0.394
Trunk anteversion angle (°)	8.8 ± 5.8	7.3 ± 2.3	0.937	8.9 ± 4.7	7.4 ± 1.1	0.485
Calf anteversion angle (°)	55.8 ± 9.0	64.6 ± 9.9	0.093	47.2 ± 24.5	48.2 ± 32.0	0.818

ROM = range of motion.

**Table 2:** Comparison of joint angles in leg cycle between roller skiing and on-snow skiing at ski/roller ski touchdown and take-off.

Angle	Ski/Roller Ski Touchdown			Ski/Roller Ski Take-Off		
	Roller Skiing	On-Snow Skiing	*p*-Value	Roller Skiing	On-Snow Skiing	*p*-Value
Hip flexion–extension angle (°)	42.4 ± 8.6	35.4 ± 16.0	0.485	−4.4 ± 9.9	−10.0 ± 5.4	0.589
Hip adduction–abduction angle (°)	−8.8 ± 4.0	−4.5 ± 5.1	0.132	−3.1 ± 3.0	−6.1 ± 3.4	0.180
Knee flexion–extension angle (°)	134.4 ± 10.0	119.8 ± 8.7	0.041 *	149.4 ± 6.2	148.4 ± 3.3	0.699
Body anteversion angle (°)	6.4 ± 2.3	15.1 ± 2.5	0.002 *	7.3 ± 2.4	13.4 ± 3.2	0.009 *
Trunk anteversion angle (°)	10.6 ± 5.8	12.8 ± 4.9	0.310	8.1 ± 1.4	12.7 ± 2.6	0.009 *
Calf anteversion angle (°)	11.8 ± 16.6	37.5 ± 14.6	0.041 *	42.1 ± 5.8	51.3 ± 2.4	0.009 *

* Significant difference (*p* < 0.05) between roller skiing and on-snow skiing.

**Table 3:** Comparison of joint ROM in pole cycle between roller skiing and on-snow skiing during poling and recovery phases.

ROM	Poling Phase			Recovery Phase		
	Roller Skiing	On-Snow Skiing	*p*-Value	Roller Skiing	On-Snow Skiing	*p*-Value
Shoulder flexion–extension angle (°)	43.9 ± 5.8	53.9 ± 12.6	0.240	53.2 ± 7.3	64.3 ± 8.6	0.093
Shoulder adduction–abduction angle (°)	57.0 ± 11.3	67.4 ± 20.8	0.699	65.6 ± 22.4	78.3 ± 16.4	0.394
Elbow flexion–extension angle (°)	55.9 ± 12.3	58.7 ± 15.8	0.937	57.3 ± 17.1	57.7 ± 16.4	0.937
Body anteversion angle (°)	4.3 ± 1.0	5.5 ± 2.0	0.485	4.7 ± 1.6	4.6 ± 0.9	0.699
Trunk anteversion angle (°)	8.4 ± 4.0	7.2 ± 1.5	>0.999	8.2 ± 4.6	7.4 ± 1.1	0.589
Pole inclination (°)	29.4 ± 5.0	32.8 ± 6.5	0.394	—	—	—

ROM = range of motion.

**Table 4:** Comparison of joint angles in pole cycle between roller skiing and on-snow skiing at pole touchdown and take-off.

Angle	Pole Touchdown			Pole Take-Off		
	Roller Skiing	On-Snow Skiing	*p*-Value	Roller Skiing	On-Snow Skiing	*p*-Value
Shoulder flexion–extension angle (°)	30.7 ± 10.6	30.4 ± 20.1	0.937	−11.0 ± 7.7	−20.5 ± 6.1	0.065
Shoulder adduction–abduction angle (°)	35.8 ± 17.4	31.3 ± 19.7	0.818	−15.9 ± 12.2	−32.4 ± 8.5	0.041 *
Elbow flexion–extension angle (°)	77.2 ± 5.8	78.7 ± 12.1	0.818	127.9 ± 13.2	134.7 ± 16.2	0.485
Body anteversion angle (°)	6.9 ± 2.0	13.0 ± 3.2	0.015 *	7.1 ± 2.2	12.9 ± 2.5	0.009 *
Trunk anteversion angle (°)	7.8 ± 2.6	12.6 ± 3.7	0.026 *	7 ± 2.3	11.4 ± 4.7	0.065
Pole inclination (°)	12.9 ± 6.3	34.1 ± 17.5	0.015 *	40.4 ± 4.3	43.9 ± 8.4	0.699

* Significant difference (*p* < 0.05) between on-snow and on-land skiing.

## Data Availability

Pseudonymized datasets are available to external collaborators subject to agreement on the terms of data use and publication of results. To request the data, please contact Mujia Ma (mamujia5683@gmail.com).
